# Oxidized Low Density Lipoprotein Induced Caspase-1 Mediated Pyroptotic Cell Death in Macrophages: Implication in Lesion Instability?

**DOI:** 10.1371/journal.pone.0062148

**Published:** 2013-04-25

**Authors:** Jing Lin, Xiling Shou, Xiaobo Mao, Jiangchuan Dong, Nilesh Mohabeer, Kishan kumar Kushwaha, Lei Wang, Yousu Su, Hongcheng Fang, Dazhu Li

**Affiliations:** 1 Department of Cardiovascular Medicine, Shaanxi Provincial People's Hospital, Xi'an, China; 2 Department of Cardiology, Institute of Cardiovascular Diseases, Union Hospital, Tongji Medical College, Huazhong University of Science and Technology, Wuhan, China; 3 Department of Cardiology, Shenzhen Sixth People's Hospital (Nanshan Hospital), Huazhong University of Science and Technology, Union Shenzhen Hospital, Shenzhen, China; UAE University, United Arab Emirates

## Abstract

**Background:**

Macrophage death in advanced lesion has been confirmed to play an important role in plaque instability. However, the mechanism underlying lesion macrophage death still remains largely unknown.

**Methods and Results:**

Immunohistochemistry showed that caspase-1 activated in advanced lesion and co-located with macrophages and TUNEL positive reaction. In in-vitro experiments showed that ox-LDL induced caspase-1 activation and this activation was required for ox-LDL induced macrophages lysis, IL-1β and IL-18 production as well as DNA fragmentation. Mechanism experiments showed that CD36 and NLRP3/caspase-1/pathway involved in ox-LDL induced macrophage pyroptosis.

**Conclusion:**

Our study here identified a novel cell death, pyroptosis in ox-LDL induced human macrophage, which may be implicated in lesion macrophages death and play an important role in lesion instability.

## Introduction

Atherosclerosis is a chronic disease characterized by accumulation of lipid within the vessel wall, inflammation, local changes in structure, and cell death [1]. Macrophage death now has been clearly confirmed as an important determinant of the fate of atherosclerotic lesion [1]–[3]. Macrophage death in late atherosclerotic lesion decreases cellularity, promoting necrotic core formation. Dying macrophage in atherosclerotic lesions leads to the release of growth factors, cytokines, proteases, and intracellular lipid into the extracellular space. These factors may serve to propagate inflammation, promote plaque disruption and arterial thrombosis, the proximate causes of acute cardiovascular events [1], [4].

Although apoptosis in atherosclerosis is well documented, unraveling contribution of apoptosis to atherosclerosis is complex, and in many cases contradictory. Results from electron microscopy studies found that in human atherosclerotic plaques the majority of dying cells had an ultrastructure typical of cells undergoing cell lysis [5]. Caspase-3, the executor caspase of apoptosis, is reported to be less expressed in advanced atherosclerotic lesion [6]. In a recent study, adoptively transferring p53−/− marrow into ApoE−/− mice and fat feeding for 12 weeks generate plaques with less apoptosis, but more macrophages, and necrotic core and doubling of lesion area [7]. Furthermore, DNA fragmentation, which was previously reported as a morphological criterion for apoptosis, has been observed in other type of cell death, such as necrosis and pyroptosis [8], [9]. These results raised question whether non-apoptotic cell death is involved in lesion macrophage death; however, the exact mechanism remains largely unknown.

Pyroptosis is a novel form of cell death which is uniquely dependent on caspase-1. Caspase-1 is not involved in apoptotic cell death and caspase-1-deficient cells respond normally to most apoptotic signals [10]. An important function of caspase-1 is to process the proforms of the inflammatory cytokines, IL-1β and IL-18, to their active forms [11]. Caspase-1 activation in macrophages infected with *Salmonella* or *Shigella* results in processing of these cytokines and death of the host cell [12], [13]. The mechanism and outcome of this form of cell death are distinctly different from these aspects of apoptosis, which actively inhibits inflammation. The morphology of pyroptosis seems a combination of both apoptotic and necrotic cells [8]. Pyroptotic cells lose their cell membrane potential and undergo DNA fragmentation and nuclear condensation and, like apoptotic cells, show positive terminal deoxynucleotidyl transferase–mediated dUTP nick end-labeling (TUNEL) staining. Pyroptosis was initially described in macrophages infected with a range of microbial infections. However, it is recently reported that pyroptosis could also be induced by non-infectious stimuli, such as carbon black nanoparticles [14].

In this study, we examined the hypothesis that pyroptosis was involved in ox-LDL induced human macrophages death. Our data demonstrated that ox-LDL could promote caspase-1 activation in human macrophages and this activation was required for ox-LDL induced human macrophage lysis, DNA fragmentation, as well as IL-1β and IL-18 production, confirming that pyroptotic cell death occurred in ox-LDL treated human macrophages and this dell death may be implicated in lesion macrophage death.

## Methods

### Ethics

This study was approved by the Ethics Committee of Tongji Medical College, Huazhong University of Science and Technology. Written informed consents were received from all patients involved in this study. The study protocol conforms to the ethical guidelines of the 1975 Declaration of Helsinki as reflected in a priori approval by the institution's human research committee.

### Tissue Specimens

Human non-atherosclerotic and atherosclerotic arteries (femoral and carotid arteries) were obtained from patients who underwent femoral and carotid endarterectomy in the Department of Vascular Surgery, Union Hospital, or from autopsy (n = 2). After surgery, tissues were halved at the site of the maximum artery diameter. One half was fixed in formalin and embedded in paraffin for histology; the other half was for protein analysis. Plaque subtypes were determined in compliance with the modified American Heart Association classification, based on morphological description, proposed by Stary et al [15]. Because one of the main goals in this study was to discriminate the overall pro-inflammatory mediators between early and advanced stages of development, we classified the plaques as follows: Type I and type II, including plaque with intimal thickenings and xanthomas, are uniformly termed early atherosclerotic lesions (EAL, n = 4), whereas type IV, type V and type VI, including atheroma, fibrolipid plaque, fibrousplaque, calcific lesion fibrotic lesion and complicated lesions, were defined as advanced atherosclerotic lesions (AAL, n = 6). The baseline characteristics of the patients are summarized in online Table S1.

### Immunohistochemistry and Immunofluorescence

Immunohistochemical analyses were carried out to identify the expression of cleaved caspase-1 in human atherosclerotic lesions. Sections were routinely stained with haematoxylin–eosin and then stained with anti-human cleaved caspase-1 (Santa Cruz, USA). The reaction products were visualized by treating the slide with 3, 30-diaminobenzidine tetra hydrochloride (DAB Liquid System, Dako) and counterstained with haematoxylin. For control, the primary antibody was replaced by PBS or by non-specific human IgG. For quantitative analysis of immunohistochemistry, plaque images were visualized and analyzed with HMIAS Series Color Medical Image Analyze System (Champion Image Ltd., China).

Double labeling staining was performed using anti-cleaved caspase (caspase-1, -3, -8 and -9) and CD68 antibody (Santa Cruz, USA), and with secondary antibodies labeled with FITC or PE (eBioscience, USA). For control, primary antibodies were replaced with non-specific immunoglobulins. Processed sections were visualized with a fluorescent microscope (Olympus Microscope BX-51, Japan) or a confocal microscope (Alsi, Nikon) and quantified using designed software.

### Preparation of LDL and Copper-oxidized LDL

Ox-LDL was prepared as described previously [16].

### Cell Culture

Peripheral blood mononuclear cells (PBMCs) from healthy donors were obtained by Ficoll density gradient centrifugation. Monocytes were separated by adhesion for 4 h at 37°C. Differentiation of adherent cells into macrophages was carried out for 7 days in RPMI 1640 supplemented with 2 mM glutamine, 100 u/ml penicillin, 100 µg/ml streptomycin, 10% FCS and 10% autologous serum. After day 7 differentiated macrophages were cultured with or without ox-LDL, lipopolysaccharides (LPS, Sigma, USA), staurosporine (STS, Sigma, USA), CD36 blocking antibody (Santa Cruz, USA), control blocking antibody (Santa Cruz, USA), or the relevant vehicle controls (PBS).

### Inhibition Experiments

For inhibition experiments, cells were pretreated with caspase inhibitor (Ac-DEVD-CHO, Ac-DEVD-CHO, Ac-LEVD-CHO, Ac-VEID-CHO, Z-IETD-CHO, Z-LEHD-CHO, or Z-VAD-CHO, Life science, Germany) at 100 µM concentrations for 30 min prior to initiation of experiment.

### RNA Interference and Transfection

Small interfering RNA (siRNA) was synthesized by RiBoBio Inc. (China). The sequences of siRNA were listed in Table S2. Human monocyte-derived macrophages (HMDMs) were transfected at 70% confluence. Transfection of siRNA was performed at a final concentration of 50 nM using Lipofectamine 2000 (Invitrogen, USA).

### Evaluation of Transfection Efficiency

HMDMs were plated in 6-well plates 24 hour prior to transfection with 50 nM Cy3-labeled siRNA as described above. Transfections were performed in triplicate for each treatment. One part was washed with PBS, fixed in paraformaldehyde 4% for 30 min in the dark, washed with methanol for 3 to 5 times, and washed again with PBS. After several washes in PBS, the cover slips were mounted on PBS/glycerin. Cells were photographed under a light or fluorescence microscope (for Cy3, wave length 555 nm; Olympus Microscope BX-51). And the other was prepared for real-time RCR to determine the inhibition rate.

### Cytokines Assay

Cultured supernatants or patients' blood serum were collected and kept frozen at −80°C until the cytokine levels (IL-1β IL -18, IL-33, IL-6, TNF-α, and MCP-1) were determined by ELISA assays according to the manufacturer’s instructions. All commercial kits were purchased from R&D Systems (USA). Each sample was tested in triplicate.

### Cell Death Assay

Cell death was assessed by various methods including lactate dehydrogenase (LDH) release and EthD-III/calcein AM staining. For LDH release, culture supernatants were collected and cytotoxicity was measured using the Cytotox 96 kit (Promega, USA) as directed by the manufacturer’s instructions. Total LDH release was achieved by adding Triton X-100 and percentage cytotoxicity was calculated as 100 × (experimental LDH − spontaneous LDH**)/(**Total LDH release − spontaneous LDH).

EthD-III/calcein AM staining (Viability/Cytotoxicity Assay Kit, Biotium, USA), which provides a two-color fluorescence staining on both live and dead cells, was also employed for cell death assay. The virtually nonfluorescent cell-permeant calcein AM is enzymically converted to the intensely fluorescent calcein, producing an intense uniform green fluorescence and EthD-III enters dead cells, thereby producing a bright red fluorescence. Death cell was achieved by counting the ratio of red positive cells to the total number of cells.

### Analysis of DNA Fragmentation

Nuclear DNA fragmentation was detected by TUNEL staining using a commercial kit (Promega, USA) according to the manufacturer’s instructions. Samples were counterstained with DAPI and analyzed with a fluorescence Microscopy. For cultured cells, TUNEL-positive nuclei (green) were determined by randomly counting 10 fields of the section and were expressed as a percentage of the total nuclei population. For plaque sections, formalin-fixed neonatal plaque tissue was permeabilized with proteinase K and counterstained with DAPI to visualize cell nuclei. Tissue sections were incubated with the TUNEL reaction mixture in the dark for 60 min at 37°C. For the quantification of apoptosis in tissue sections, the number of TUNEL-positive cells was normalized to the total number of cells.

### Caspase Activity

Caspase activity was measured by using colorimetric assay. Briefly, cells were lysed in lysis buffer containing 50 mM HEPES, at pH 7.4, 5 mM, 3-[(3-cholamidopropyl)-dimethylammonio]-1-propanesulfonate, and 5 mM dithiothreitol. Nuclei and organelles were removed by centrifugation at 20,000 *g* and 50 µg of total cytosolic protein was used to assess cytosolic caspase activity. To this end, cell homogenates were incubated up to 4 hours at 37°C with corresponding caspases substrate conjugated to the chromophore *p*-nitroanilide (Ac-YVAD-*p*NA; Ac-VDVAD-*p*NA; Ac-DEVD-*p*NA; Ac-LEVD-*p*NA; Ac-VEID-*p*NA; z-IETD-*p*NA; and Ac-LEHD-*p*NA). Cleavage of substrate by corresponding caspase releases *p*NA, which was quantified spectrophotometrically at 405 nm using an enzyme-linked immunosorbent assay reader (BioRad, USA). All caspases substrate were purchased from Enzo Life Sciences International Inc. (Germany).

### Measurement of Intracellular Reactive Oxygen Species (ROS) Levels

The level of intracellular ROS was determined on the basis of the oxidative conversion of cell permeable 20,70-dichloro fluorescein diacetate (DCFH-DA, Sigma, USA) to fluorescent dichloro fluorescein (DCF) upon reaction with hydroxyl radical, hydrogen peroxide, or peroxynitrite. Briefly, cells were incubated with control media (PBS) or ox-LDL in the presence or absence of antioxidants (N-acetylcysteine or vitamin C) for 18 hours. Then cells were washed twice with cold PBS (pH 7.4) and incubated with DCFH-DA at room temperature for 30 min in dark. Fluorescent signal was recorded by using a fluorescence microscopy (488 nm filter; Olympus Microscope BX-51, Japan). The fluorescence intensity of eight fields per dish was measured and the reactive oxygen species (ROS) level was quantified by measurement of fluorescence intensity with HMIAS-2000 software. Three parallel experiments were performed. Results were shown as the mean value.

### Gene Expression Analysis

Total RNA from cultured cells was isolated using Trizol reagent (Takara Biotechnology, Japan) according to the manufacturer’s instructions. cDNA was synthesized using RNA PCR Kit (Takara Biotechnology, Japan) and used as PCR template. Quantitative PCR was performed on ABI PRISM 7900 Sequence Detector system (Applied Biosystems, USA) using SYBR Green I Assay (Takara Biotechnology, Japan). GAPDH was used as endogenous control. Relative gene expression level (the amount of target, normalized to endogenous control gene) was calculated using the comparative Ct method formula 2-DDCt. The sequences of primers for PCR were listed in Table S3.

### Western Blot Analysis

Equal protein amounts were loaded onto SDS-PAGE gels. After running gels, proteins were transferred onto nitrocellulose membranes. Membranes were blocked in 5% nonfat milk and primary antibody incubations were performed with 3% BSA (overnight at 4°C). Antibodies used were anti-caspase-1 (Santa Cruz, USA), anti-cleaved caspase (Santa Cruz, USA), anti-IL-1β (Santa Cruz, USA), anti-IL-18 (Santa Cruz, USA), ICAD (Santa Cruz, USA). Then membranes were incubated with peroxidase-conjugated secondary antibodies at room temperature for 2 hours. Specific band was detected with chemiluminescence assay (ECL detection reagents, Pierce) and recorded on x-ray film. Designed software was used to quantify the intensities of bands.

### Statistical Analysis

Results are shown as mean ± SEM of at least three independent experiments. The significance of differences was estimated by ANOVA followed by Student–Newmann–Keuls multiple comparison tests. *P*<0.05 was considered significant. All statistical analyses were performed with SPSS software (version 11.0).

## Results

### 1. Caspase-1 Activated in Human Advanced Atherosclerotic Plaques

**Figure 1:**
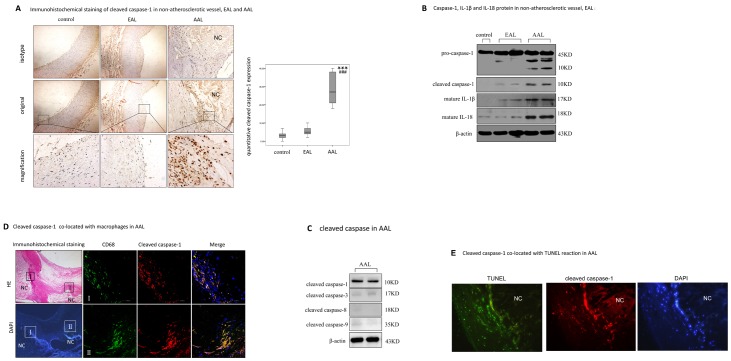
Cleaved caspase-1 over-expressed in human atherosclerotic plaque. A. Immunohistochemical analysis of cleaved caspase-1 in non-atherosclerotic vessel (control), EAL and AAL. In the left column, representative immunostaining for cleaved caspase-1(brown, arrow) in exemplary sections of non-atherosclerotic vessel (control), early atherosclerotic lesion (EAL) and advanced atherosclerotic lesion (AAL) were shown. High-power photomicrograph (×200) exhibited strong cleaved caspase-1 staining in AAL, as shown in the upper boxed area in low-power photomicrograph (×40). In the right column, bar graphs showed the relatively quantitative cleaved caspase-1 expression in control, EAL and AAL. *indicated vs control, ***p<0.001; ^#^indicated vs EAL, ^###^ *p*<0.001. NC, necrotic core. B. Western blot analysis of caspase-1, mature IL-1β and IL-18 protein. Protein was extracted from 5 vessel tissues obtained from non-atherosclerotic vessel (control), EAL and AAL. β-actin was used for protein loading controls. C. Double staining of cleaved caspase-1 co-located with CD68 positive cells in AAL. In the right column, high-power photomicrograph (×400) showed strong cleaved caspase-1 staining (red) correlated with CD68 positive cell (green) around necrotic core (NC), as shown in the boxed area of HE staining and DAPI staining (blue) in the left column (×40). D. Double staining of cleaved caspase-1(red) co-located with TUNEL reaction (green) in AAL (×200).

To explore whether caspase-1 activated in atherosclerotic lesion, we stained human atherosclerotic sections with cleaved caspase-1, an activated form of caspase-1 to examine its expression Exemplary findings of activated caspase-1 in normal vessel, EAL and AAL plaques (Figure 1A) are shown. Quantitative analysis showed that compared with normal vessels and EAL, increased cleaved caspase-1 was strongly detected in AAL plaques (p<0.001). Western blotting consolidated that caspase-1 was activated in advanced plaques (Figure 1B), as shown by the appearance of 10KD cleaved caspase-1 as well as by mature IL-1β and IL-18. Figure 1C showed that compared with the strong appearance of cleaved caspase-1, we hardly found an obvious cleavage of apoptotic caspase (caspase-3, -8 and -9) in AAL. Immunofluorescence staining confirmed that this abundant cleaved caspase-1 in AAL was most notably co-located in the macrophage-rich layer around the necrotic core (Figure 1D), as well as co-located within TUNEL-positive reaction (Figure 1E). However, we did not detect a co-location of TUNEL reaction with cleaved apoptotic caspase (caspase-3, -8 and -9) (Figure S1). These results suggested that caspase-1 was activated in advanced atherosclerotic plaques and may be involved in lesion macrophage death.

### 2. Ox-LDL Promotes Caspase-1 Activation in Human Macrophages

To unravel the mechanism of caspase-1 activation in advanced atherosclerotic plaques, we stimulated human monocyte-derived macrophages with native LDL or ox-LDL, or cultured alone. Cells were treated with LPS (1 µg/ml) followed by ATP 30 min was as a positive control for caspase-1 activation [9], [17].

**Figure 2:**
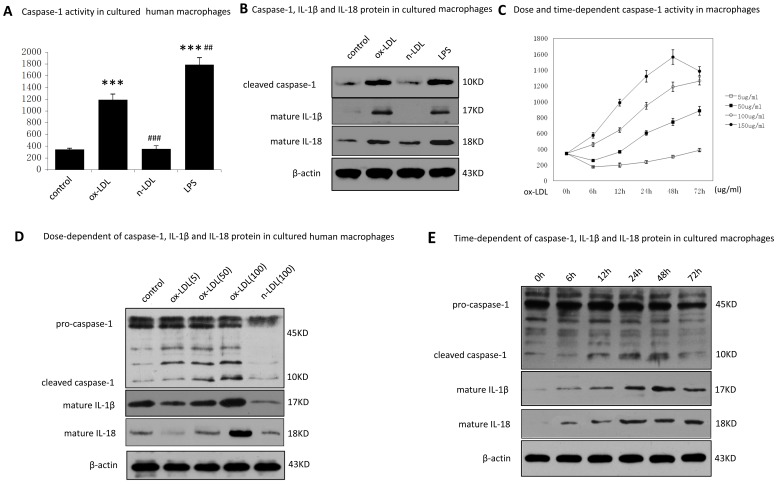
Caspase-1 activated in ox-LDL induced human macrophages. Human macrophages were cultured with PBS (control), with ox-LDL (100 µg/ml) or with native LDL (100 µg/ml) for 48 hours. After incubation, caspase-1 activity was measured by Ac-YVAD-*p*NA (A). Cells cultured with LPS (1 µg/ml) for 6 h followed by ATP (5 mM) for 30 min as a positive control. Data are presented as mean±SEM of at least three independent experiments. *indicated vs untreated cells (control), ***p<0.001. ^#^indicated vs ox-LDL treated cells, ^##^ *p*<0.01, ^###^ *p*<0.001.*NS*, not significant differences (*p*>0.05). B. Protein of cleaved caspase-1, mature IL-1β and IL-18 in cell lysates was analyzed by western blot. β-actin was used for protein loading controls. C. Dose and time-dependent caspase-1 activity in HMDMs. Ox-LDL (5 µg/ml, 50 µg/ml, 100 µg/ml, and 200 µg/ml) were incubated with HMDMs for indicated time, and then caspase-1 activity was measured by Ac-YVAD-*p*NA. Data are presented as mean±SEM of at least three independent experiments. D. Ox-LDL ((5 µg/ml, 50 µg/ml, 100 µg/ml)) or native LDL (100 µg/ml) were incubated with HMDMs for 48 h, then protein of caspase-1, mature IL-1β and IL-18 protein in cell lysates were measured by western blot. β-actin was used for protein loading controls. E. Ox-LDL(100 µg/ml) were incubated with HMDMs for indicated duration, then cleaved caspase-1, mature IL-1β and IL-18 protein in cell lysates were measured by western blot. β-actin was used for protein loading controls.

Caspase-1 activation was measured using the specific colorimetric caspase-1 substrate, Ac-YVAD-*p*NA and Western-blot. Figure 2A showed that compared with naive LDL, ox-LDL (100 µg/ml) promoted a significantly increase in caspase-1 activity, up to 3.4 fold in human macrophages. Using antibodies specific against the p10 subunit of caspase-1, we confirmed caspase-1 activated in the cell lysates (Figure 2B). Furthermore, matured IL-1β and IL-18 can also be detected in ox-LDL treated cell lysates. To obtain further details of caspase-1 activation in ox-LDL treated human macrophages, both time and dose-response experiments were performed. We found that ox-LDL promoted a dose and time dependent caspase-1 activation in HMDMs (Figure 2C). Similar results were obtained from Western blot analysis, as shown by the increasing appearance of the 10KD cleaved caspase-1 and matured IL-1β and IL-18 proteins in cell lysates (Figure 2D and 2E).

### 3. Caspase-1 Activation was Required for ox-LDL Induced Human Macrophage Lysis

**Figure 3:**
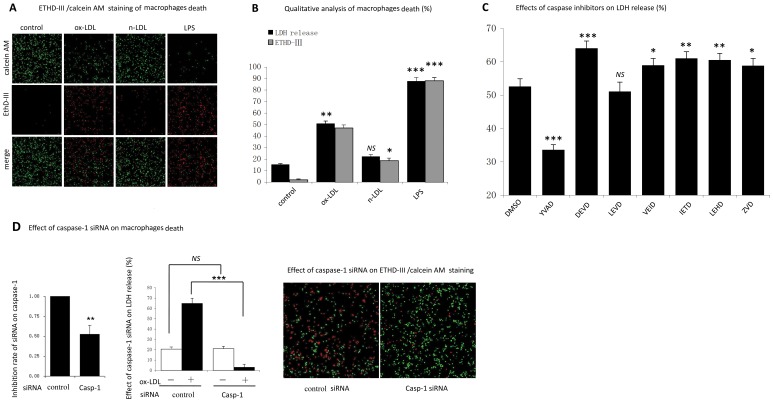
Activated caspase-1 was required for ox-LDL induced macrophages death. A. Cell was cultured as described previously. Cells death was visualized by ETHD-III (red) and calcein AM (green) staining (×100). B. Qualitative analysis of cell death expressed as a percentage of LDH release by Triton X-100 detergent or a percent of ETHD-III positive cells in the total cells. *indicated vs untreated cells, *p<0.05; **p<0.01; ***p<0.001. *NS*, not significant differences (*p*>0.05). C. Cells were pretreated for 1 hour with vehicle (DMSO), with inhibitors of caspase-1 (Ac-YVAD-CHO), caspase-3 (Ac-DEVD-CHO), caspase-4 (Ac-LEVD-CHO), caspase-6 (Ac-VEID-CHO), caspase-8 (Z-IETD-CHO), caspase-9 (Ac-LEHD-CHO), or with pan-caspase (z-VAD-CHO), and then cells were cultured with ox-LDL (100 µg/ml) for 48 h. Cell death was determined by LDH release. Inhibitors concentration was 100 µM in all cases. *indicated vs cells pre-cultured with DMSO.*p<0.05; **p<0.01; ***p<0.001. *NS*, not significant differences (*p*>0.05). D. Cells were transfected with non-targeting siRNA (control) or siRNA specific for caspase-1. After transfection, inhibition rate of siRNA was measured by RT-PCR. Transfected cells were cultured for 48 h with or without ox-LDL (100 µg/ml). Cell death was determined by LDH release and ETHD-III/calcein AM staining (×100). ***p<0.001. *NS*, not significant differences (*p*>0.05). Data are presented as mean±SEM of at least three independent experiments.

Cell death in our study was measured in two separate assays: LDH release and EthD-III/calcein AM staining. We found that compared with naive LDL, either ox-LDL or LPS induced a strong cell lysis, as shown by the increased LDH release and extensive EthD-III positive cells (Figure 3A and B). Consistent with previous study, our results also found that ox-LDL induced macrophages in a dose-dependent manner.

Next, to further investigate whether caspase-1 was involved in ox-LDL induced macrophage death, inhibitory experiment was carried out. Our results showed that although apoptotic caspase (caspase-3, caspase-6, caspase-8 and caspase-9) were activated in ox-LDL treated cells (Figure S2), none of these corresponding inhibitors could block ox-LDL-induced cell lysis (Figure 3C). In contrat, caspase-1 inhibitor showed a strong suppression in cell lysis after exposure to ox-LDL. Caspase-1 inhibitor reversed LDH release in ox-LDL treated from 55.6±2.3% to 33.6±1.5% (p<0.001). To obtain more direct evidence for the involvement of caspase-1 in ox-LDL induced macrophage death, we targeted caspase-1 in human macrophages with siRNA. Figure 3D showed that compared with non-targeting siRNA, caspase-1 specific siRNA exhibited a strong protection on ox-LDL induced HMDMs death, as shown by the presence of less EthD-III- positive cells in the whole cell population.

### 4. Caspase-1 was Required for Cytokines Production in ox-LDL Treated Human Macrophages

**Figure 4:**
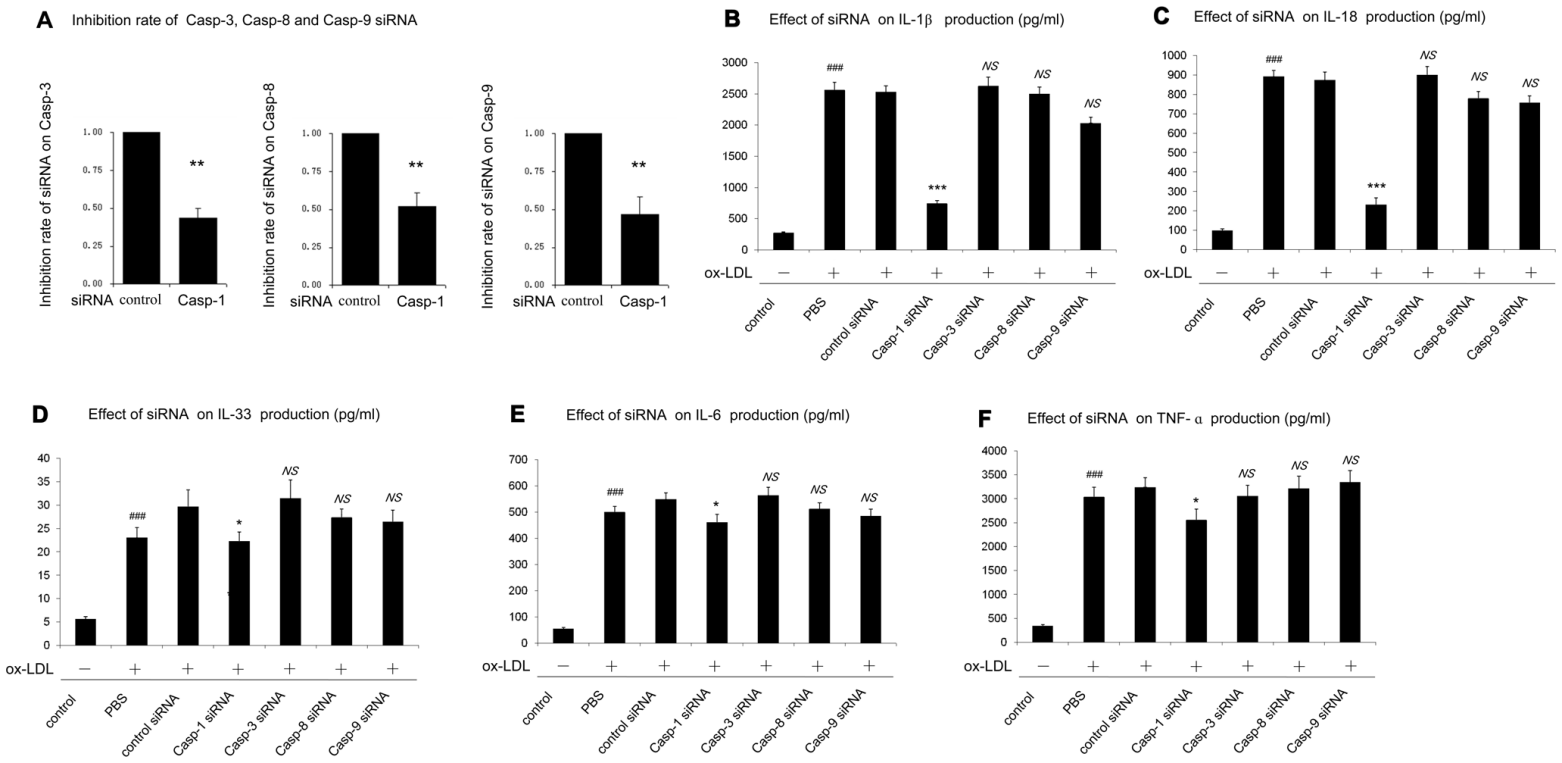
Caspase-1 activation was required for cytokines production. Cells were transfected with non-targeting siRNA (control), siRNA specific for caspase-1, caspase-3, caspase-8 and caspase-9. After transfection, cells were cultured for 48 h with or without ox-LDL (100 µg/ml). Cytokines production of IL-1β, IL-18, IL-33, TNF-α, IL-6 and MCP-1 was determined by ELISA. *p<0.05; ***p<0.001. *NS*, not significant differences (*p*>0.05). Data are presented as mean±SEM of at least three independent experiments.

Previously, our results showed that mature proteins of IL-1β and IL-18 were increased in ox-LDL induced HMDMs lysates. Here, our results showed that a significant secretion of IL-1β and IL-18 cytokines was detected in culture supernatant. Furthermore, cytokines of IL-33, IL-6, TNF-α and MCP-1, which have been implicated in the downstream response of caspase-1, were also increased in ox-LDL induced human macrophages (Figure 4).

To determine whether caspase-1 was involved in these increased cytokines in human macrophages, inhibitory experiment with inhibitors as well as with siRNA was employed. Figure 4A showed that compared with non-targeting siRNA, caspase-1 specific siRNA exhibited a statistically significant inhibition on IL-1β, IL-18 IL-33, IL-6 and TNF-α cytokines production. A significant decrease in IL-33, IL-6, and TNF-α cytokines was also found in caspase-1 knockout human macrophages (p<0.05). However, we did not detect a significant inhibition in cells transfected with specific siRNA for caspase-3, caspase-8 nor caspase-9 (Figure 4). Also, a similar trend of these cytokines was obtained in the parallel inhibitory experiment using the specific caspase-1 inhibitor and corresponding apoptotic caspase inhibitors (inhibitors for caspase-3, caspase-8 and caspase-9) (Data not shown).

### 5. Caspase-1 Involved in ox-LDL Induced DNA Fragmentation in Human Macrophages

Pytoptotic cell death is characterized by caspase-1 dependent cell lysis, inflammation, and DNA fragmentation. We previously had described caspase-1 was required for ox-LDL induced macrophage lysis and inflammatory cytokines production. Thus, we next exam whether caspase-1 was required for ox-LDL induced macrophage DNA fragmentation. DNA fragmentation in our study was assessed using TUNEL reaction. Human macrophages cultured with staurosporine (STS) were employed as a pattern of apoptosis while cells cultured with LPS and ATP as a model of pyroptosis.

**Figure 5:**
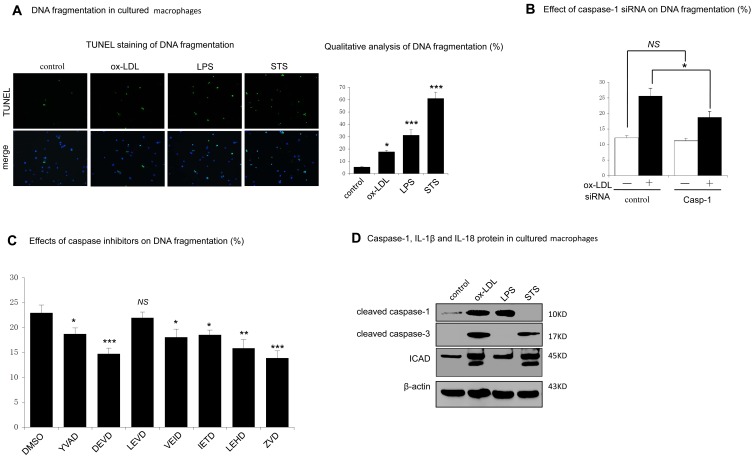
Involvement of activated caspase-1 in ox-LDL induced DNA fragmentation. A. Cells were cultured with PBS (control), with ox-LDL (100 µg/ml, 48 h), LPS (1 µg/ml, 6 h) followed by ATP (5 mM, 30 min), or with STS (1 Μm, 4 h).After incubation, DNA fragmentation was measured by TUNEL staining (green). Qualitative analysis of DNA fragmentation by randomly counting 10 fields of the section and were expressed as a percentage of the total nuclei population. * indicated vs untreated cells (control), *p<0.05; ***p<0.001. B. Cells were transfected with non-targeting siRNA (control) or siRNA specific for caspase-1. Then cells were cultured for 48 h with or without ox-LDL (100 µg/ml). DNA fragmentation was determined by TUNEL positive counting. *p<0.05. *NS*, not significant difference (*p*>0.05). C. Cells were pretreated for 1 hour with vehicle (DMSO) or with corresponding caspase inhibitors (100 uM), and then cells were cultured with ox-LDL (100 µg/ml) for 48 h. DNA fragmentation was determined by TUNEL positive counting. *indicated vs control, *p<0.05; **p<0.01; ***p<0.001. *NS*, not significant difference (*p*>0.05). D. Protein of cleaved caspase-1 and ICAD in cell lysates were measured by western blot. β-actin was used for protein loading controls. Data are presented as mean±SEM of at least three independent experiments.

Figure 5A showed TUNEL positive reaction was observed in ox-LDL induced HMDMs. However, this fragmented DNA could be a result of apoptotic caspase mediated apoptosis or caspase-1 mediated pyroptosis or other form of cell death. With siRNA, we found that targeting siRNA for caspase-1 could largely block DNA fragmentation in ox-LDL treated human macrophages (Figure 5B). With specific caspase inhibitors, however, we found that both apoptotic inhibitors (caspase-3, caspase-6 and caspase-9) and caspase-1 inhibitor had a suppressive effect on ox-LDL induced DNA fragmentation (Figure 5C), suggesting that both pyroptotic way and apoptotic way be involved in ox-LDL induced DNA fragmentation. This assumption was further consolidated by proteolysed inhibitor of caspase-activated DNase (ICAD) in both LPS/ATP and ox-LDL induced macrophages, but not in STS induced macrophage death (Figure 5D).

### 6. Role of Inflammasome Components ASC and NLRP3 in ox-LDL Induced Pyroptosis in Human Macrophages

**Figure 6:**
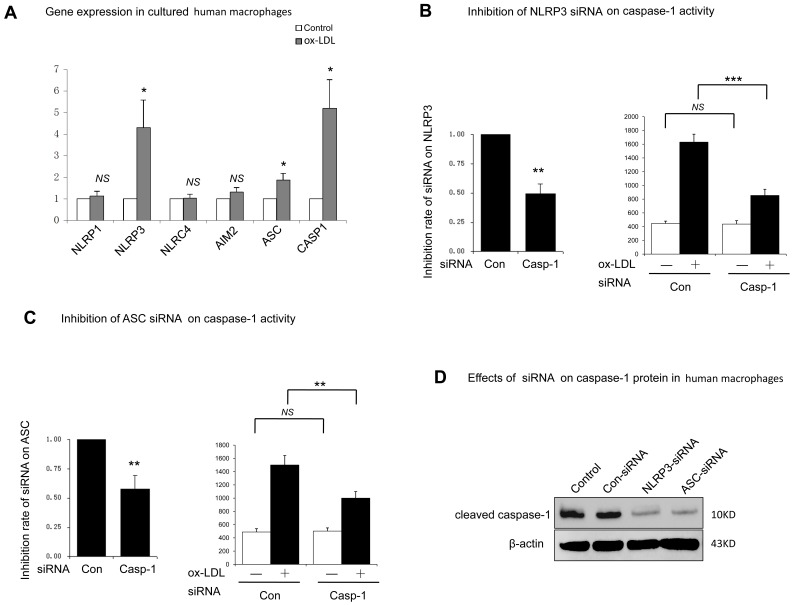
Role of inflammasome components ASC and NLRP3 in ox-LDL induced caspase-1 activation. A. Cells were cultured with PBS (control) or with ox-LDL (100 µg/ml, 48 h). Gene expression of inflammasome components was measured by RT-PCR. * indicated vs control, *p<0.05. *NS*, not significant differences (*p*>0.05). Cells were transfected with non-targeting siRNA (control) or siRNA specific for NLRP3 (B) or ASC (C). After transfection, inhibition rate of siRNA was measured by RT-PCR. Transfected cells were then cultured for 48 h with or without ox-LDL (100 µg/ml, 48 h). Caspase-1 activity was measured by Ac-YVAD-*p*NA. ***p<0.001. *NS*, not significant differences (*p*>0.05). D. Cells were transfected and cultured as described above. Protein of cleaved caspase-1 was measured by western blot. Un-transfected human macrophages cultured with ox-LDL was used as control. β-actin was used for protein loading controls. Data are presented as mean±SEM of at least three independent experiments.

Inflammasomes are multi-protein complexes formed by several members of the NOD-like receptor (NLR) family, procaspase-1, and adaptor protein ASC. To data, four inflammasomes, including NLRP1, NLRP3, NLRC4, and AIM2 were reported. Our results showed that inflammasome components, including ASC, caspase-1, NLRP1, NLRP3, NLRC4, and AIM2, were all expressed in human macrophages (Figure 6A). However, only up-regulated NLRP3, caspase-1 and ASC transcripts could be observed in ox-LDL treated human macrophages.

The role of NLRP3 and ASC in ox-LDL induced pyroptosis was further determined by the RNA interference experiment. Consistent with previous reports that NLRP3 or ASC deficient mice macrophages had a defective caspase-1 activation and IL-1β production response to cholesterol crystal, our results showed that NLRP3 and ASC knockdown macrophages had a significant inhibition on caspas-1 activation (Figure 6B and C). Protein analysis also confirmed that NLRP3 inflammsome was required for caspase-1 activation in ox-LDL treated macrophages (Figure 6D).

### 7. Involvement of CD36 and ROS in ox-LDL Induced Pyroptosis in Human Macrophages

**Figure 7:**
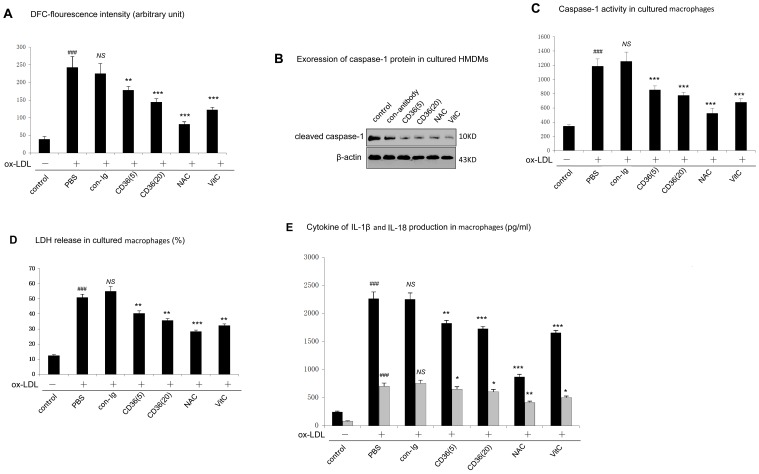
Involvement of CD36 in ox-LDL induced caspase-1 activation, LDH release and IL-1β and IL-18 production. A. Cells were pretreated with vehicles (control), NAC (10 mM, 24 h) or VitC (100 mM, 24 h), or with CD36 blocking antibody (5 ug/ml or 20 ug/ml), then cells were cultured with ox-LDL (100 µg/ml, 48 h). Intracellular ROS in HMDMs were assessed by DCFH2-DA. B. Cleaved caspase-1 was measured by western blot. β-actin was used for protein loading controls. C. Caspase-1 activity was measured by Ac-YVAD-*p*NA. ^###^p<0.001. *NS*, not significant differences (*p*>0.05). D. Cell death was measured by the percentage of LDH release. E. Cytokine production of IL-1β and IL-18 was measured by ELISA. *indicated vs cells cultured with ox-LDL, *p<0.05; **p<0.01; ***p<0.001. ^#^indicated vs cells cultured with control. ^###^ *p*<0.001. *NS*, not significant differences (*p*>0.05). Data are presented as mean±SEM of at least three independent experiments.

Recent studies demonstrated that ROS generation is a mechanism for NLRP3 inflammasome activation [18]. Thus, we next explore whether a similar mechanism apply to NLRP3 inflammasome activation occurred in ox-LDL treated human macrophages. Our results showed that ox-LDL treatment promoted robust ROS generation (Figure 7A); and pretreatment of cells with antioxidant, vitamin C or N-acetylcysteine (NAC) inhibited ox-LDL induced caspase-1 activity (Figure 7B and C), cell lysis (Figure 7D) as well as IL-1β and IL-18 production (Figure 7E). These results indicated that NLRP3/caspase-1 activation in ox-LDL treated human macrophages via generating ROS.

CD36 is the major receptor of ox-LDL on human macrophages surface. It was recently suggested that CD36 could induce ROS generation in murine vascular smooth muscle cells. [19] To examine if CD36 was implicated in ROS generation in ox-LDL treated human macrophages, we incubated human macrophages with CD36 blocking antibody before adding with ox-LDL. Compared with non-blocking control antibody, CD36 blocking antibody showed a strong inhibition on ROS generation, caspase-1 activity, cell lysis and cytokines production (Figure 7), suggesting CD36 was involved in ox-LDL induced pyroptosis in human macrophages.

## Discussion

In the current study, we provide evidence to identify a novel form of cell death, caspase-1 dependent pyroptosis, in ox-LDL induced human macrophages. To our knowledge, this is the first demonstration of this form of cell death in ox-LDL induced human macrophages, which may provide new perspective to understand cell death in atherosclerotic lesions.

Despite the widespread use of the apoptosis-versus-necrosis paradigm, many other forms of cell death exist, including autophagy, oncosis, and pyroptosis [20]. Whereas apoptosis is an active, programmed process of autonomous cellular dismantling that avoids eliciting inflammation, pyroptosis is an inflammatory cell death, companied with abundant pro-inflammatory cytokines production. While features of apoptosis include activation of apoptotic caspase (caspases-3, -8, and -9), distinctive DNA cleavage as well as nuclease [20], features of pyroptosis include capase-1 dependent cell lysis, inflammatory cytokines production and DNA fragmentation [21].

Cell death in advanced lesion has been confirmed to play an important role in plaque instability. Although apoptosis in atherosclerosis is well documented, our study found that cleaved apoptotic caspase was rarely expressed in advanced lesion. Furthermore, TUNEL positive reaction in AAL was not co-located with these apoptotic caspase. These finding strongly indicated that apoptosis was not the major form of cell death in atherosclerosis, especially in advanced lesion.

Oxidation of low density lipoprotein (ox-LDL), which is abundantly deposited in local vessels, has been confirmed to be implicated in the pathogenesis of atherosclerosis due to its cytotoxicity for all cells implicated in atherogenesis. Many studies have described that human monocyte-derived macrophage exposed to ox-LDL express many morphological features characteristic of apoptotic cell death [22]. However, features including loss of membrane integrity, eliciting inflammation, and cell organ damage, which do not exist during apoptosis, were also reported in ox-LDL treated human macrophages [23]. Lactate dehydrogenase (LDH) release from damaged cells indicates the occurrence of loss of cell membrane integrity. LDH release and inflammatory cytokines secretion in our study confirmed these non-apoptotic features occurred in ox-LDL treated human macrophages. Although several groups proposed that defective clearance and post-apoptotic necrosis was responsible for these non-apoptotic features [24], results from our own and others contradict with this assumption [23]. It could be imaged if cell completely died in a post-apoptotic necrosis manner, apoptotic caspase inhibitors should be able to reverse cell lysis or inflammatory cytokines production, even partly. However, we and others found that inhibitors of apoptotic caspase (caspase-3, -8 and -9) failed to block any cell lysis and inflammatory cytokines production in ox-LDL treated human macrophages [23], suggesting non-apoptotic pathway may be involved in ox-LDL induced macrophages death.

In this study, we found ox-LDL induced a dose and time-dependent caspase-1 activation. Furthermore, the inductive caspase-1 activation was required for ox-LDL induced macrophage lysis. This possibly due to caspase-1-dependent pore formation in the plasma membrane [25], which led to potassium efflux, osmotic pressure, water influx, cell swelling and necrosis-like lysis [25]. Another important function of caspase-1 in our study is processing IL-1β and IL-18 to their active forms [11]. The mechanism of IL-1β and IL-18 secretion required two independent signals: signals provided by NF-κB activators to transcriptionally and translationally up-regulate pro -IL-1β and pro-IL-18 and second, the activation of caspase-1 by inflammasomes [26], [27]. We previously had reported ox-LDL could serve as TLR ligand, activating NF-kB pathway and promoting inflammation. [16]. Here, we confirmed that ox-LDL could initiate caspase-1 activation, indicating that ox-LDL could be sufficient to provide both signals 1 and 2 needed to activate IL-1β and IL-18 release from cells. Caspase 1 activation, however, is required for production of inflammatory cytokines more than IL-1β and IL-18. Caspase-1 in our study was also required for IL-33, TNF-α and IL-6 production. While IL-33, IL-1β and IL-18 may employ the similar mechanism to cleave, TNF-α and IL-6 release could be due to caspase-1 mediated cleavage of the TLR adapter protein TIRAP (Toll/interleukin-1 receptor domain-containing adapter protein) [28].

Degradation of chromosomal DNA is a terminal event in the life of a cell and has been served as a criterion of apoptosis. However, recent search suggested DNA fragmentation also could observed in pyroptosis. The TUNEL positivity of Salmonella and Shigella infected macrophages initially led to the assumption that the cell death induced by these bacteria was apoptosis [12], [13]. However, this cell death was eventually confirmed as pyroptosis by caspase-1 dependence. Our results showed that both apoptotic caspase (caspase-3, -8 and -9) and inflammatory caspase-1 had an inhibitory effect on DNA fragmentation, implying that both pyroptotic way and apoptotic way involved in ox-LDL induced macrophage DNA fragmentation. This assumption was further consolidated by ICAD cleavage in LPS/ATP induced pyroptosis and ox-LDL induced macrophages, but not in STS induced apoptosis.

There have been a number of inflammasomes responsible for caspase-1 activation. Upon activation, inflammsomes auto-cleave pro-caspase-1 into the active p10/p20 tetramer, allowing processing and activation of cytokines IL-1β and IL-18 as well as pyroptosis [26], [27]. NLRP3 inflammasome is mostly understood to be activated by a range of microbial infections. However, it has been found that it could also be activated by non- infectious stimuli, such as silica, and uric acid crystal [29], [30]. Our study showed transcription of these four inflammasomes in human macrophages. However, only NLRP3 inflammasome transcription was increased in human macrophages after exposure to ox-LDL, suggesting that the NLRP3 inflammasome pathway was involved in ox-LDL induced caspase-1 activation. This result was further supported by the RNA inference experiment in which NLRP3 or ASC knockdown macrophages were defective in caspase-1 activation.

CD36, known as the major receptor for ox-LDL, is important for lipid uptake and macrophage-derived foam cell formation. However, the role of CD36 is far more than forming foam cells. It is suggested CD36 could regulate apoptotic cell clearance, initiate signal transduction, and serve as pattern recognition receptors for promoting inflammation [31], [32]. CD36 transcription and protein synthesis were significantly up-regulated when macrophages were exposed to ox-LDL [33]. Our results showed that CD36 blocking antibody could inhibit pyroptic cell death in ox-LDL treated human macrophage with inhibition on caspase-1 activation, cell lysis as well as IL-1β and IL-18 production. Our results also showed that the mechanism for CD36 involvement in ox-LDL induced pyroptosis was through ROS generation. It is demonstrated that ROS could be a sensor for NLRP3 activation [27], [34]. As such, our data suggested that ox-LDL could act with CD36, generating ROS and activating the NALP3 inflammasome in human macrophages as well.

In conclusion, our study here identified a novel cell death, pyroptosis in ox-LDL induced human macrophage. The impetus for this study was also to explore pathways that may be involved in promoting macrophage death in atherosclerotic lesion. Considering the abundant caspase-1 activity was observed in advanced lesions and more closely related to lesion macrophage death, it is speculated that pyroptosis may be implicated in lesion macrophage death and inflammation, and therefore may play a critical role in plaque instability.

## Supporting Information

Figure S1 Expression of TUNEL reaction and cleaved caspase in AAL. Immunofluorescence staining of TUNEL reaction (green) and cleaved caspase (caspase-3, -8 and -9) (red) and DAPI (blue) in AAL (×100).
(TIF)

Figure S2 Caspase activity in cultured macrophages. Human macrophages were cultured with PBS (control), with ox-LDL (100 µg/ml, 48 h), LPS (1 µg/ml, 6 h) followed by ATP (5 mM, 30 min), or with STS (1 Μm, 4 h). Activity of caspase was measured by corresponding substrates. * indicated vs control. *p<0.05; ** *p*<0.01; ***p<0.001. Data are presented as mean±SEM of at least three independent experiments.
(TIF)

Table S1 **Baseline characteristics of the patients.**
(DOCX)

Table S2 **The sequences of siRNAs used in this study.**
(DOCX)

Table S3 **The sequences of primers for real time RT-PCR used in this study.**
(DOCX)
