# Supplementary figures and images for: Oxidized Low Density Lipoprotein Induced Caspase-1 Mediated Pyroptotic Cell Death in Macrophages: Implication in Lesion Instability?
Source: PLoS One. 2013 Apr 25;8(4):e62148. doi: 10.1371/journal.pone.0062148 (PMC3636212; doi:10.1371/journal.pone.0062148)

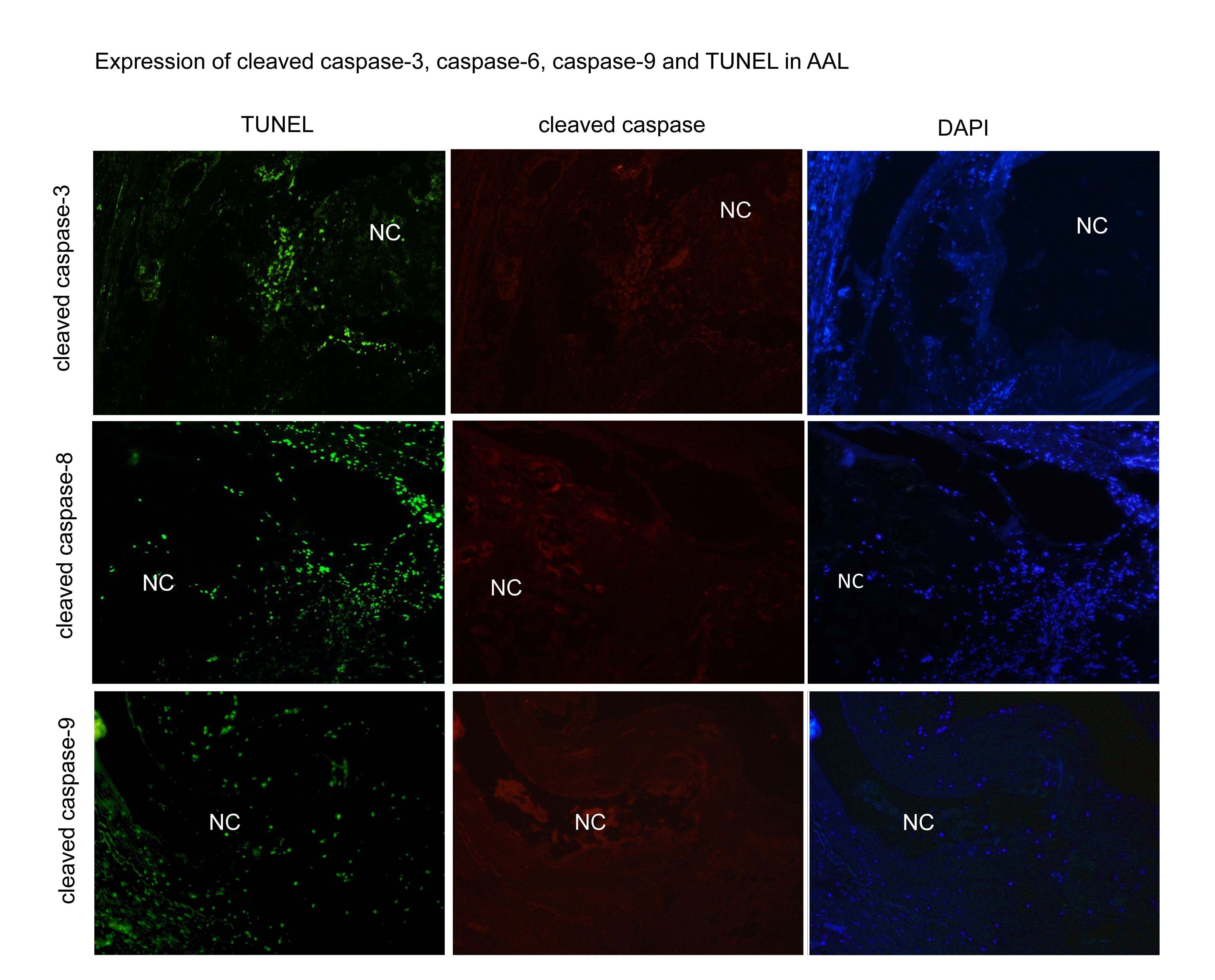

Supplement: Figure S1 — Expression of TUNEL reaction and cleaved caspase in AAL. Immunofluorescence staining of TUNEL reaction (green) and cleaved caspase (caspase-3, -8 and -9) (red) and DAPI (blue) in AAL (×100). (TIF) [file pone.0062148.s001.tif]

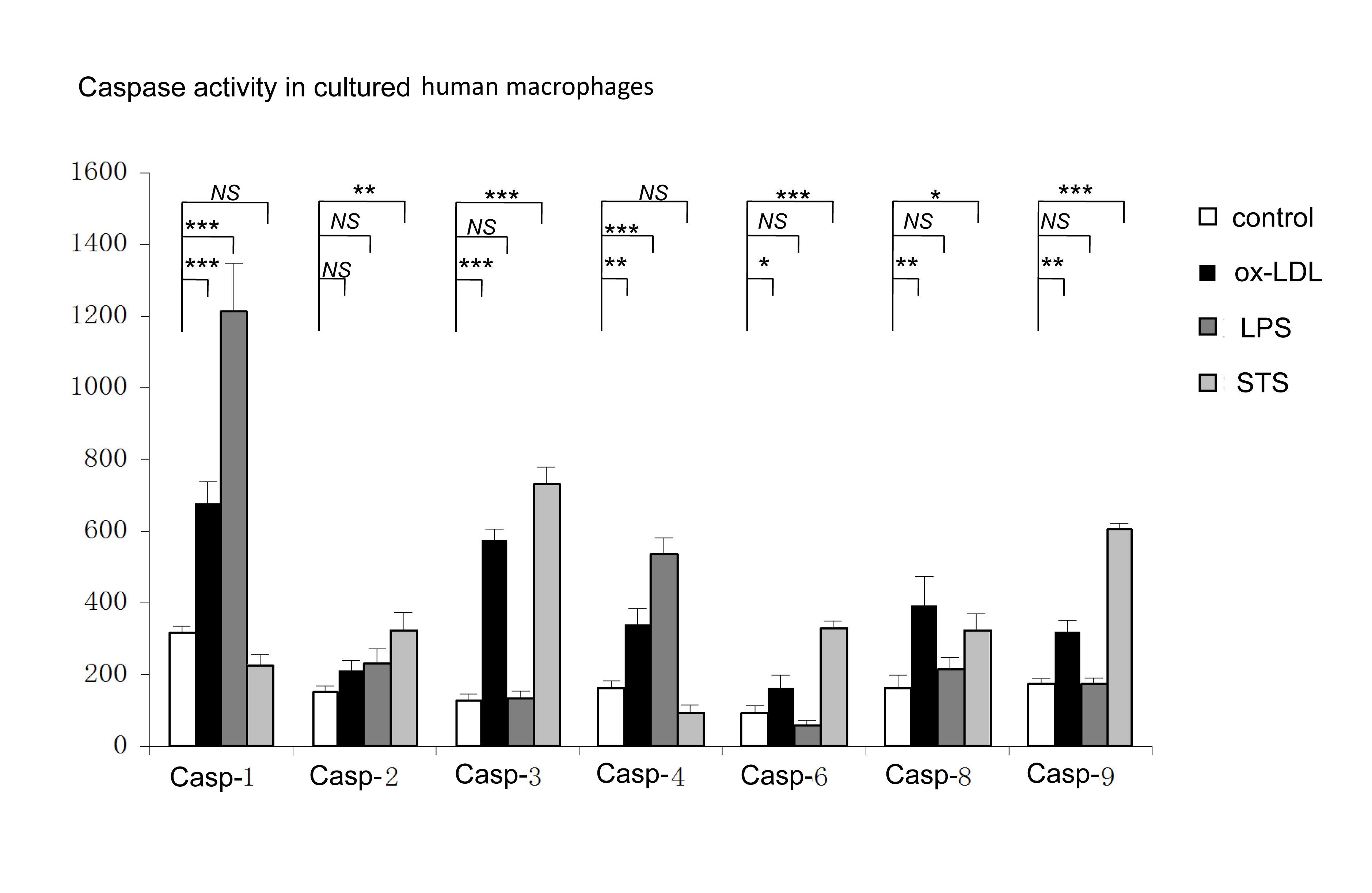

Supplement: Figure S2 — Caspase activity in cultured macrophages. Human macrophages were cultured with PBS (control), with ox-LDL (100 µg/ml, 48 h), LPS (1 µg/ml, 6 h) followed by ATP (5 mM, 30 min), or with STS (1 Μm, 4 h). Activity of caspase was measured by corresponding substrates. * indicated vs control. *p<0.05; ** p<0.01; ***p<0.001. Data are presented as mean±SEM of at least three independent experiments. (TIF) [file pone.0062148.s002.tif]
