# Supplementary material for: Oxidized Low Density Lipoprotein Induced Caspase-1 Mediated Pyroptotic Cell Death in Macrophages: Implication in Lesion Instability?
Source: PLoS One. 2013 Apr 25;8(4):e62148. doi: 10.1371/journal.pone.0062148 (PMC3636212; doi:10.1371/journal.pone.0062148)
Supplement: Table S1 — Baseline characteristics of the patients. (DOCX) [file pone.0062148.s003.docx]

**Supplemental Table 1. Baseline characteristics of the patients**

Patients, n 12

Age, y 53±14

Male/sex, % 58.3

Diabetes mellitus, % 33.3

Systemic hypertension, % 75

Hyperlipoproteinemia, % 58.3

Smoking, % 50

History of myocardial infarction, % 25
