# Supplementary material for: Oxidized Low Density Lipoprotein Induced Caspase-1 Mediated Pyroptotic Cell Death in Macrophages: Implication in Lesion Instability?
Source: PLoS One. 2013 Apr 25;8(4):e62148. doi: 10.1371/journal.pone.0062148 (PMC3636212; doi:10.1371/journal.pone.0062148)
Supplement: Table S2 — The sequences of siRNAs used in this study. (DOCX) [file pone.0062148.s004.docx]

**Supplemental Table 2. siRNA**

siRNA Sense Antisense

Casp-1 GGUUCGAUUUUCAUUUGAG CUCAAAUGAAAAUCGAACC

Casp-3 CCCACUUCUUGUAUGCAUA UAUGCAUACAAGAAGUCGG

Casp-8 GCUCUUCCGAAUUAAUAGA UCUAUUAAUUCGGAAGAGC

Casp-9 CGACCUGACUGCCAAGAAA UUUCUUGGCAGUCAGGUCG

ASC GAUGCGGAAGCUCUUCAGU ACUGAAGAGCUUCCGCAUC

NLRP3 GGUGUUGGAAUUAGACAAC GUUGUCUAAUUCCAACACC

control UUCUCCGAACGUGUCACGU ACGUGACACGUUCGGAGAA
