# Supplementary material for: Oxidized Low Density Lipoprotein Induced Caspase-1 Mediated Pyroptotic Cell Death in Macrophages: Implication in Lesion Instability?
Source: PLoS One. 2013 Apr 25;8(4):e62148. doi: 10.1371/journal.pone.0062148 (PMC3636212; doi:10.1371/journal.pone.0062148)
Supplement: Table S3 — The sequences of primers for real time RT-PCR used in this study. (DOCX) [file pone.0062148.s005.docx]

**Supplemental Table 3. Primers**

Primer Forward Reverse

Casp-1 TCCAATAATGGACAAGTCAAGCC GCTGTACCCCAGATTTTGTAGCA

NLRP1 GGAGGCCTTGGTGAAACC CGATGTCACTCGGGCTATCA

NLRP3 GGAGAGACTTTATGAGAAAGCAA GCTGTCTTCCTGGCATATCACA

NLRC4 AGAAAGGATCTCTGCATGAAGGT GCGGTCCAAATGGTCACATTC

AIM2 CAGAAGCACAAGCGTTTACAGA CTCTTTGCTATCTCCAGGCCA

GADPH ACCCACTCCTCCACCTTTG CTCTTGTGCTCTTGCTGGG
